# Organizational health literacy and health promotion in health care settings: Results from Germany

**DOI:** 10.1093/eurpub/ckac131.360

**Published:** 2022-10-25

**Authors:** K Rathmann, LD Salewski, LV Zelfl

**Affiliations:** 1 Department of Health Sciences, Fulda University of Applied Sciences, Fulda, Germany; 2 Public Health Centre Fulda, Fulda University of Applied Sciences, Fulda, Germany

## Abstract

**Introduction:**

Health care organizations (HCO) are increasingly required to provide health literate structures and processes to strengthen organizational health literacy (OHL). So far, little is known about the extent of OHL in HCO in Germany. Aims are therefore 1) to examine the level of OHL in health care organizations, and 2) to investigate the impact of organizations level of health prevention and workplace health promotion on the level of OHL.

**Methods:**

Analyses are based on a nationwide cross-sectional study (online survey) among management staff (N = 564) in HCO (hospitals, nursing homes, facilities for disabled people). OHL was measured by the German version of the ‘Health literate health care organization 10 item questionnaire’ (HLHO-10). Health prevention and workplace health promotion were operationalized by the “Worksite Health Promotion Capacity Instrument’ (WHPCI). Uni- and bivariate analyses were carried out, on an item-basis and as an index (median-split).

**Results:**

Regarding OHL, 55.1 % of the health care organizations reported below-average levels of OHL. On an item-basis, the highest below-average levels were given for the standard ‘communication’ (59.1 %) and the ‘provision of information to patients/residents via various media’ (57.4 %). Regarding the level of health prevention (51.8 %) and the existence of workplace health promotion structures (55.7 %), more than half of HCO reported a below-average level. In addition, results showed that HCO that indicate a below-average level of prevention and few structures of workplace health promotion also revealed a below-average level of OHL.

**Conclusions:**

There is need to strengthen OHL in German HCO.

**Key messages:**

• HCO are required to strengthen HLO, particular in communication and participatory approaches.

• A higher level of prevention and workplace health promotion on HCO can contribute to the implementation of OHL initiatives.

